# Evaluation of the Pulp Space Morphology of the Mandibular Premolars in a Greek Population by Cone Beam Computed Tomography

**DOI:** 10.3390/dj14080526

**Published:** 2026-08-18

**Authors:** Konstantinos Koutoulas, Nikos Pantazis, Vasileios Makris, Dimitrios Tsatsas, Alexandros Skountzouris, Eleftherios Terry R. Farmakis

**Affiliations:** 1 Private Practice, 20100 Corinth, Greece; konktls@gmail.com; 2 Department of Hygiene, Epidemiology and Medical Statistics, School of Medicine, National and Kapodistrian University of Athens, 11527 Athens, Greece; npantaz@med.uoa.gr; 3 Private Practice, 9000 Ghent, Belgium; vasilismakris93@gmail.com; 4 Private Practice, 10674 Athens, Greece; microendoathens@gmail.com; 5 Private Practice, 93350 Paris, France; aiskountzouris@gmail.com; 6 Department of Endodontics, Dental School, National and Kapodistrian University of Athens, 11527 Athens, Greece

**Keywords:** mandibular premolar, pulp space anatomy, cone beam computed tomography, CBCT, Greek descent population

## Abstract

**Background/Objectives**: This study aims to evaluate the pulp space morphology (PSM) of both first and second mandibular premolars in a sample of a Greek population using cone beam computed tomography (CBCT). **Methods**: Two hundred and ninety nine CBCTs of a Greek population were assessed to evaluate the PSM using Vertucci’s classification. CBCTs were prescribed for non-endodontic reasons. **Results**: In the aforementioned data set, the most common PSM was Vertucci’s 1 (V1) in all subgroups of mandibular premolars. V3 was the second most frequent PMS in the #34 group (10.67%), and it was V5 in the #35 group (3.1%), V3 in the #44 group (14%), and V8 in the #45 group (2.7%), with the rest of the morphologies being sparse. Across the four teeth, there was a statistically significant difference in Vertucci class distribution (*p* = 0.001). No significant difference was found between genders (*p* = 0.120); bilateral symmetry analysis showed high concordance for both premolars. **Conclusions**: Within the limitations of this study, it is concluded that the reported pulp space morphology in the mandibular premolars of a Greek sub-population sample presented as less complicated compared to other similar reports of (sub)-populations of different descent.

## 1. Introduction

The success rate of endodontic therapy is reported to range from 45 to 95% and is dependent on many individual factors [1]. An influential factor with significant contribution in the endodontic therapy outcome is the root canal morphology [2]. Its variations from regularity may jeopardize the therapeutic outcome [3,4]. Thus, knowledge of pulp space anatomy—not only the most common form, but also its variations—is clinically mandatory [5]. Moreover, a lack of knowledge of pulp space anatomy irregularities can adversely affect the success of therapy [6]. Several methodologies have been applied to study the pulp space configuration [7].

Retro-alveolar radiographs (or periapical x-rays) have been a valid tool for preoperative assessment of each case. By taking these radiographs from different angles, according to the technique described by Clark, the presence of two independent canals can be verified [8]; however, this two-dimensional approach produces limited information by superimposition of anatomical structures, a constraint that CBCT imaging overcomes.

Many researchers support the belief that the mandibular premolars present the greatest difficulty of all teeth to perform successful endodontic treatment because of the numerous variations in RC morphology; thus, their pre-op evaluation is crucial [9,10].

Currently, cone beam computed tomography (CBCT) is the imaging modality with the greatest impact in Endodontics [11,12,13,14], as it enables the 3D visualization of the root canal system. It helps to identify the number of roots and root canals and their convergence or divergence from each other, as described by Vertucci in 1984 [3].

This study was designed to evaluate the diversity of the root canal morphology (based on Vertucci’s Classification) of mandibular first and second premolars in a sample of a Greek population by using CBCT. To the best of the authors’ knowledge, based on a search of PubMed and Scopus using the terms “Vertucci classification”, “mandibular premolars”, “CBCT”, and “Greek population”, no prior CBCT-based study has evaluated the root canal morphology of mandibular premolars in a Greek population, and the present study aims to address this gap in the literature. This study will provide insights into the complexity and diversity of root canal morphology and will allow for better comprehension of root canal system anatomy and, subsequently, improved quality of endodontic treatment, if needed.

## 2. Materials and Methods

### 2.1. Subjects

Due to the retrospective design of the study, the enrolled subjects were chosen from a series of CBCT examinations recruited from the database of an imaging center in Greece, performed from October 2025 to April 2026. These were patients who were referred by dentists or oral and maxillofacial surgeons for (a) evaluation of mandibular pathology, (b) facial trauma, or (c) complex pre-surgical implant treatment planning. All examinations were coded when handed to the authors, without any identification data included, besides gender. The study was conducted in accordance with the Declaration of Helsinki, and the protocol was approved by the Ethics Committee of the Athens Dental Association, Athens, Greece (project identification code: 1690/2026).

Out of 568 CBCT scans, a total of 299 CBCT data sets defined this study‘s cohort based on the following inclusion criteria: (a) CBCT examinations should include the mandibular premolars, (b) scans should not present movement and/or stripe artifacts, and (c) the patients were of Greek nationality, as recorded in the referral information accompanying each examination.

Teeth with previous root canal treatment, incomplete root development, root resorption, extensive caries, restoration related artifacts, developmental root abnormalities, or pathological conditions that could interfere with canal identification or teeth of non-Greek patients were excluded.

### 2.2. CBCT Imaging

All examinations were performed with a Kodak CS 9300 CBCT imaging unit (Carestream Health, Rochester, NY, USA) at 110 kV and 2–4 mA depending on the patient’s head (average 18.4 mAs/patient), with an exposure time of 3.6 s. A reconstruction volume of 15 cm × 15 cm and a voxel size of 0.3 mm was used. Reconstructions of the volumetric dataset were created by using the manufacturer’s proprietary software (CS 3D Imaging software, v3.5.7.0, Carestream Health Inc., Rochester, NY, USA). These examinations were acquired for non-endodontic reasons and is why the resulting voxel size and spatial resolution are lower than those in several CBCT studies referred to in the Discussion; this is addressed as a limitation.

### 2.3. Image Evaluation

The retrospective evaluation of the volumetric data sets was independently performed by two observers (V. M and K. K) which were previously calibrated over 10 CBCTs ordered for complex endodontic anatomy (Cohen‘s Kappa == 0.9238). The observers were blinded regarding the patients‘ medical and dental history (clinical, histological and radiographic). Additionally, no prior individual information, which might have biased the examiners’ opinions, could be obtained by the observers because of the applied codification.

The image analysis was standardized and performed in all the multiplanar reconstructions (axial, coronal and sagittal) of each volume sequentially from the apex to the crown. Additionally, contrast and brightness were varied to achieve optimal visualization of the anatomy of the root canal system of lower premolars. We attempted to reach equilibrium in order to avoid any false positive findings as a result of image noise or artifact. The number of RCs, following Vertucci’s classification, of the teeth under investigation were recorded. Discordant classifications between the two observers were resolved by joint re-reading of the volumetric dataset and consensus agreement.

### 2.4. Statistical Analysis

All analyses were performed using Stata 18 (StataCorp, College Station, TX, USA). The units of analysis were the tooth for overall descriptive analyses and the participant for contralateral symmetry analyses. Categorical variables are presented as counts and percentages. Differences in the distribution of Vertucci classifications across tooth types and between genders were assessed using Fisher’s exact test.

For the evaluation of bilateral symmetry, analyses were restricted to participants with both contralateral teeth available (#34–#44 and #35–#45 pairs). Agreement between left and right teeth was quantified as the proportion of concordant pairs (% agreement).

Symmetry of Vertucci classifications between contralateral teeth was assessed using the symmetry test for matched categorical data. For sparse tables, exact symmetry *p*-values were calculated. A two-sided *p*-value < 0.05 was considered statistically significant.

No adjustment for clustering was applied when participants contributed more than one contralateral pair, as symmetry analyses were conducted separately by tooth pair.

The frequencies of the number of roots and the number of root canals of the teeth included in the sample were recorded and analyzed with descriptive statistics. Where the overall Fisher’s exact test for tooth type differences was significant, post hoc pairwise Fisher–Freeman–Halton exact tests were performed for comparison of each of the six pairs, with a Bonferroni-corrected significance threshold (α = 0.05/6 = 0.0083).

## 3. Results

**Figure 1:**
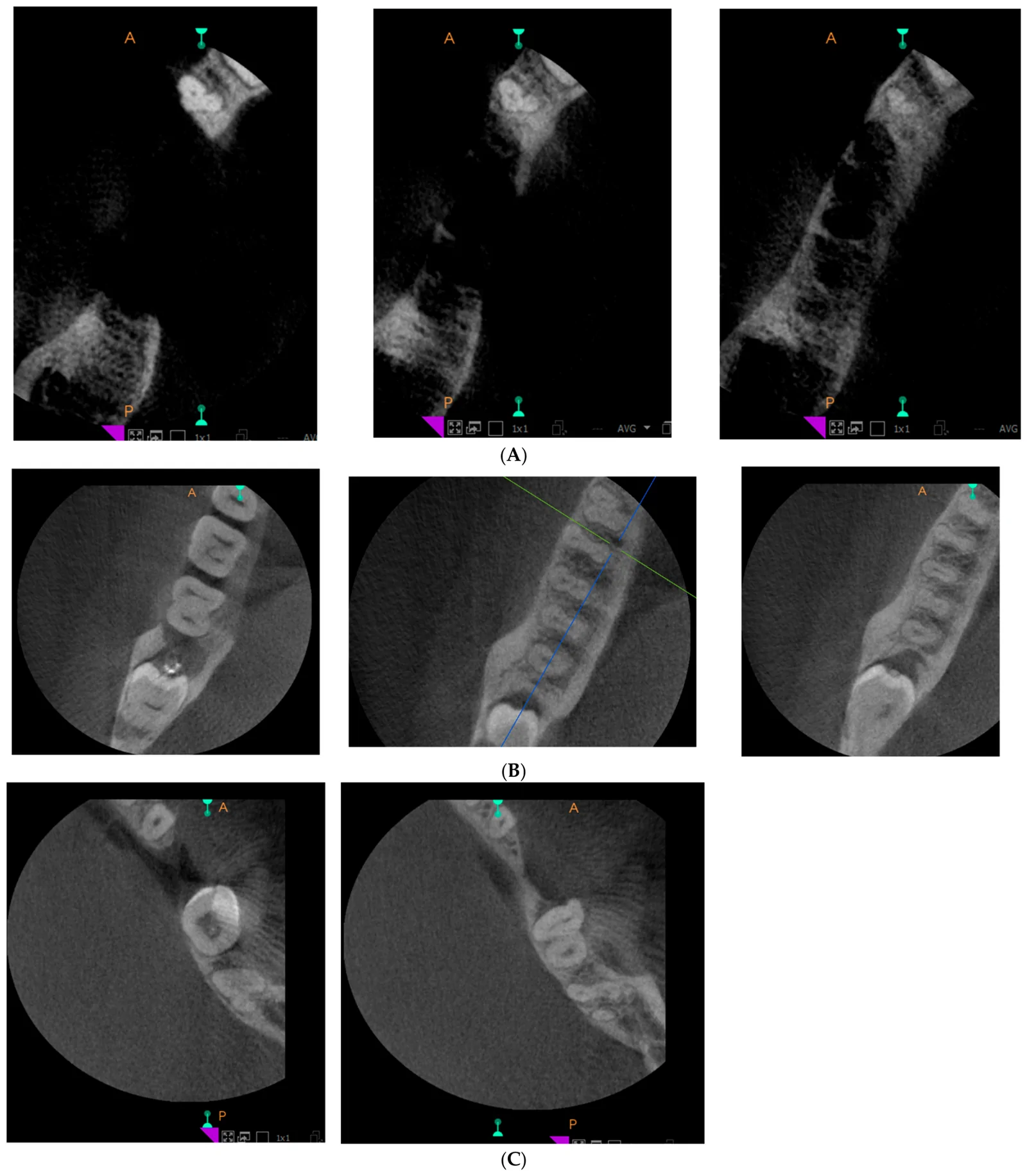
(**A**) Case #34 with two roots, Vertucci’s classification V4. (**B**) Case #45 with three roots, Vertucci’s classification V8. (**C**) Case #44 with one root, Vertucci’s classification V1.

Of the 299 examined CBCTs, premolars were finally evaluated on the Vertucci’s classification as shown in Table 1. All premolars exhibited V1 as their main morphology (one root with one root canal). In more detail, sixty four (85.3%) mandibular left first premolars (#34) were found to present PSM classified as Vertucci’s V1, with the second most frequent (10.7%) being the V3 variation. One canal divides into two sections in the root, and then merges to one canal (or 1-2-1). Regarding the mandibular left second premolars (#35), sixty two (95.4%) were found to present V1 morphology, with the other variations being rare. Furthermore, a total of 70 (81.4%) mandibular right first premolars (#44) were found to present V1 morphology, with the second most common being V3 (14.0%). And finally, 69 (94.5%) mandibular right second premolars (#45) were found to present as V1, with the other variations being rare. Representative images can be seen in Figure 1.

Pulp space morphology differences between males and females were observed, as shown in Table 2. Of the examined CBCTs, 124 (93.2%) and 141 (84.9%) were found to present V1, with just 5.3% being obtained from males and 8.4% presenting with a V3 morphology, respectively.

In more detail, in the male subgroup, both #34 and #44 presented an almost equal percentage (around 14.6%) of V3 variation, whereas the other variations were negligible. For the females, #44 presented with 12.82% of V3 morphology. Interestingly enough, in females, #45 presented with 100% V1 configuration. The aforementioned data are shown in Table 3 and Table 4.

Vertucci type 1 was the predominant canal configuration across all mandibular premolars. A statistically significant difference in morphology distribution was observed across tooth types, whereas no significant difference was detected between genders. More specifically, the first premolars comprised a more diverse internal anatomy, notably types III and V of the Vertucci classification. Post hoc pairwise Fisher–Freeman–Halton exact tests (Bonferroni-corrected α = 0.0083) showed that this overall difference was driven mainly by #44 versus #35 (*p* = 0.002) and #44 versus #45 (*p* = 0.004), both statistically significant after correction; the #34 vs. #35, #34 vs. #45, #34 vs. #44, and #35 vs. #45 comparisons did not reach the corrected significance threshold.

Type I canal configuration was significantly more prevalent in mandibular second premolars. Types II, VI and VIII were rare. No significant difference was detected between genders. One important remark is that no V4 variation was identified in this sample. It is important to mention that that was the case, and not an accidental omission of data.

Complete bilateral concordance (100% agreement) was observed for mandibular first premolars (#34–#44), indicating perfect symmetry in the studied sample, as shown in Table 5. For mandibular second premolars (#35–#45), agreement was high (94.1%), with only one discordant pair identified (Table 6). The symmetry test did not demonstrate a statistically significant difference between left and right second premolars, supporting overall bilateral morphological symmetry.

These findings suggest a strong tendency toward contralateral symmetry in mandibular premolar canal morphology, particularly for first premolars, although the small paired sub-samples limit the strength of this conclusion.

## 4. Discussion

Endodontic treatment management depends on radiographic imaging in order to assess the root canal anatomy and its surrounding tissues [15]. Anatomic noise and three-dimensional evaluation are very important in endodontics [16].

CBCT scanners are categorized into those which capture the entire maxillofacial skeleton, those that capture only the maxilla or mandible, and limited-volume scanners which capture two or three individual teeth [14]. Sonick et al. reported the three-dimensional geometric accuracy superiority of CBCT against periapical radiographs [17] and no distortion or superimposition of overlying anatomy [14].

According to Vertucci, 70% of mandibular first premolars usually have one root with one root canal, and 97.5% of mandibular second premolars usually have one root with one root canal [3]. The findings of this study differ regarding the percentage of morphology in the mandibular first premolar group.

There have been numerous studies evaluating the internal anatomy of mandibular premolars of different ethnic groups. In an Israeli population, mandibular first premolars comprised Vertucci type I in 78% of the teeth examined, while second premolars comprised 97% [18]. In a Chinese population, mandibular first premolars were observed to have one root canal in 54% and complicated canals in 46% [19]. In a western Chinese population, mandibular first premolars were observed to have one root in 98% and one root canal in 87.1%, and mandibular second premolars were all observed to have one root, with 97.2% of them having one canal [20]. In an Iranian population, mandibular first premolars appeared to have one root in 86.4%, and 68.1% had one root canal, while mandibular second premolars had one root in 95% of cases and one root canal in 80.6% [21]. In an Iranian sub-population from Golestan Province, mandibular first premolars were observed to have one root in 78.5% of cases and two roots in 20.8%, while among the single-rooted first premolars, 66.9% had one canal, 31.5% had two canals, and 1.5% had three canals [22]. In a Korean study, mandibular first and second premolars had one root in 99.9% and 99.4% of cases, respectively [23], and another study found one canal with one foramen in 85.7% of first premolars and 99.5% of second premolars [24]. A Turkish population had mandibular first and second premolars with one root and one root canal in 94.2% and 98.9% of cases, respectively [25]. The Turkish Cypriot population appeared to have one root with one root canal in 92.8% of the examined mandibular premolars [26].

The second premolars in a Saudi population were observed to have one root in 98.3% of cases [27], which is similar with the results of Al-Zubaidi et al., who studied a Saudi sub-population and reported single-rooted second premolars in 99.2% of cases with 91.1% having V1 classification and reported single-rooted mandibular first premolars in 95.5% with 70% having V1 classification [28]. Similarly, another study reported one root in 100% of mandibular second premolars, with one root canal in 96.8% of cases, and 99.5% of mandibular first premolars had one root with one root canal in 69.5% and two root canals in 29.5% [29]. A further study from the Saudi western region also reported one root in first and second premolars in 94.9% and 98% of cases, respectively, with one root canal in 81.4% of mandibular first premolars and 93.2% of mandibular second premolars [30].

An Emirati sub-population showed mandibular premolars with root canal anatomy different than the one expected. Mandibular first premolars were observed to have one root in 77.3% and were C-shaped in 22.7%, while mandibular second premolars were observed to have one root in 97% and were C-shaped in 3% [31]. In a south Egypt population, first premolars were reported to have one root in 85.4% and one root canal in 57.8% of cases, and second premolars had one root in 96.5% and one root canal in 90.8% [32]. In a Thai population, mandibular second premolars showed mostly (98%) Vertucci I classification, while first premolars showed this classification in 63.1% of cases, with 28.5% classified as Vertucci type V [33]. In a Benghali sub-population from Bangladesh, first premolars had one root and one canal in 99% and 75% of cases, respectively [34]. In an Italian population, single roots were found in 97% of cases and one canal in 73.95%, while second premolars had one root in 99% of cases and one root canal in 94.12% [35]. In a Polish population, mandibular first premolars mostly had one root (97.5%), and 80.8% had one canal [36]. In our study on the Greek population, mandibular first and second premolars presented with a V1 configuration in 83.35% and 94.95% of cases, respectively.

The aforementioned percentages may differ across populations due to true biological/genetic variation, sample size differences, age distribution differences, or, most importantly, differences in CBCT protocol resolution and field of view between studies. Many comparator studies used dedicated small-FOV endodontic CBCT units, while this study’s images were acquired for pathology/trauma/implant indications, as mentioned. Given the internally contradictory voxel size description identified above, this potential confound is arguably the single most important scientific point in this report.

According to a review by Cleghorn et al., mandibular first premolars have a high incidence of two or more root canals at a percentage of 24.2%, and mandibular second premolars have two or more canals at 9% [37], which was not seen in this study. Despite the fact that mandibular first premolars are found to present greater internal variability in the reviews, mandibular second premolars are mainly found in the literature as significant/impressive case reports [38].

One parameter that has not been investigated (both in this paper and in other works) is age and its influence on the complexity of the PSM over time. It is expected to increase, due to the continuous deposition of dentin, throughout the life of pulp.

Last but not least, as the exception verifies the rule, of the two left first premolars [39,40], three right, and four left mandibular second premolars [41,42,43,44,45,46,47] reported in the literature with four root canals, two of them (both left second premolars) were found in Greek patients [Appendix A, Table A1].

In this paper, in contrast with the other premolar internal anatomy studies, the small sample size may affect the results. However, the randomness of the sample and the non-endodontic origin of the CBCTs make the results reliable. The findings and their implications should be discussed in the broadest context possible. Future research directions may also be highlighted.

## 5. Conclusions

Within the limitations and under the conditions of this study, we conclude that the tendency of lower premolars to present with complex anatomy in our sample is lower than that reported in the literature. Still, infrequent pulp space configurations were encountered.

## Figures and Tables

**Table 1:** Vertucci class across all teeth in the sample (all records)—by tooth.

Variable	34	35	44	45	Overall	*p*-Value
	*n = 75 (25.1%)*	*n = 65 (21.7%)*	*n = 86 (28.8%)*	*n = 73 (24.4%)*	*N = 299 (100%)*	
Vertucci class						0.001
*V* *1*	64 (85.3%)	62 (95.4%)	70 (81.4%)	69 (94.5%)	265 (88.6%)	
*V* *2*	0 (0.0%)	1 (1.5%)	1 (1.2%)	0 (0.0%)	2 (0.7%)	
*V* *3*	8 (10.7%)	0 (0.0%)	12 (14.0%)	1 (1.4%)	21 (7.0%)	
*V* *5*	2 (2.7%)	2 (3.1%)	2 (2.3%)	0 (0.0%)	6 (2.0%)	
*V* *6*	1 (1.3%)	0 (0.0%)	0 (0.0%)	0 (0.0%)	1 (0.3%)	
*V* *7*	0 (0.0%)	0 (0.0%)	0 (0.0%)	1 (1.4%)	1 (0.3%)	
*V* *8*	0 (0.0%)	0 (0.0%)	1 (1.2%)	2 (2.7%)	3 (1.0%)	

**Table 2:** Vertucci classifications across all teeth in the sample (all records)—by gender.

Variable	Female	Male	Overall	*p*-Value
	*n = 133 (44.5%)*	*n = 166 (55.5%)*	*N = 299 (100%)*	
Vertucci class				0.120
*V1*	124 (93.2%)	141 (84.9%)	265 (88.6%)	
*V2*	0 (0.0%)	2 (1.2%)	2 (0.7%)	
*V3*	7 (5.3%)	14 (8.4%)	21 (7.0%)	
*V5*	1 (0.8%)	5 (3.0%)	6 (2.0%)	
*V6*	1 (0.8%)	0 (0.0%)	1 (0.3%)	
*V7*	0 (0.0%)	1 (0.6%)	1 (0.3%)	
*V8*	0 (0.0%)	3 (1.8%)	3 (1.0%)	

**Table 3:** Actual numbers and relative percentages of PSM in the male premolar group.

Males	V1 No (%)	V2 No (%)	V3 No (%)	V4 No (%)	V5 No (%)	V6 No (%)	V7 No (%)	V8 No (%)	Total
**#34**	34 (82.32%)	0	6 (14.63%)	0	1 (2.43%)	0	0	0	**41**
**#35**	36 (92.3%)	1 (2.56%)	0	0	2 (5.12%)	0	0	0	**39**
**#44**	36 (76.5%)	1 (2.12%)	7 (14.89%)	0	2 (4.25%)	0	0	1 (2.12%)	**47**
**#45**	35 (89.74%)	0	1 (2.56%)	0	0	0	1 (2.56%)	2 (5.12%)	**39**
**Total**	**141**	**2**	**14**	**0**	**5**	**0**	**1**	**3**	**166**

**Table 4:** Actual numbers and relative percentages of PSM in the female premolar group.

Females	V1 No (%)	V2 No (%)	V3 No (%)	V4 No (%)	V5 No (%)	V6 No (%)	V7 No (%)	V8 No (%)	Total
**#34**	30 (85.71%)	0	2 (5.71%)	0	1 (2.85%)	1 (2.85%)	0	0	34
**#35**	26 (100%)	0	0	0	0	0	0	0	26
**#44**	34 (87.17%)	0	5 (12.82%)	0	0	0	0	0	39
**#45**	34 (100%)	0	0	0	0	0	0	0	34
**Total**	124	0	7	0	1	1	0	0	133

**Table 5:** Distribution of Vertucci classifications in contralateral mandibular first premolars (*N* = 30; teeth 34 and 44). Agreement (%) = 100.0 (30/30 pairs). Symmetry test (exact) *p*-value: >0.999.

Variable	1	3	Overall
	*n = 25 (83.3%)*	*n = 5 (16.7%)*	*N = 30 (100%)*
Left tooth Vertucci			
*V1*	25 (100.0%)	0 (0.0%)	25 (100.0%)
*V3*	0 (0.0%)	5 (100.0%)	5 (100.0%)

**Table 6:** Distribution of Vertucci classifications in contralateral mandibular second premolars (*N* = 17; teeth 35 and 45). Agreement (%) = 94.1 (16/17 pairs). Symmetry test (exact) *p*-value: >0.999.

Variable	1	3	Overall
	*n = 16 (94.1%)*	*n = 1 (5.9%)*	*N = 17 (100%)*
Left tooth Vertucci			
*V* *1*	16 (94.1%)	1 (5.9%)	17 (100.0%)

## Data Availability

The original contributions presented in this study are included in the article. Further inquiries can be directed to the corresponding author.

## Abbreviations

The following abbreviations are used in this manuscript:

CBCT	Cone Beam Computed Tomography
PSM	Pulp Space Morphology

## Appendix A

**Table A1:** Case reports of four rooted mandibular premolars found in the literature.

Authors	1st Premolars	2nd Premolars
Farmakis [42]		4 roots #35
Al-Fouzan [43]		4 root canals #35
Rhodes [44]		4 root canals #35
Ghiasi et al. [45]		4 root canals #45
Tzanetakis et al. [46]		4 root canals #35
Vaghela and Sinha [39]	4 canals #34	
Holtzman L. [41]		4 root canals #45
Hu P et al. [40]	4 root canals #34	
Niavarzi S et al. [47]		4 root canals #45

## References

[1] Friedman S., Lost C., Zarrabian M., Trope M. (1995). Evaluation of Success and Failure after Endodontic Therapy Using a Glass Ionomer Cement Sealer. J. Endod..

[2] Wang Y., Zheng Q., Zho X., Tang L., Wang Q., Zheng G., Huang D. (2010). Evaluation of the Root and Canal Morphology of Mandibular First Permanent Molars in a Western Chinese Population by Cone-Beam Computed Tomography. J. Endod..

[3] Vertucci F. (1984). Root canal anatomy of the human permanent teeth. Oral Surg. Oral Med. Oral Pathol..

[4] Sert S., Bayirli G.S. (2004). Evaluation of the Root Canal Configurations of the Mandibular and Maxillary Permanent Teeth by Gender in the Turkish Population. J. Endod..

[5] Skidmore A.E., Bjorndal A.M. (1971). Root canal morphology of the human mandibular first molar. Oral Surg. Oral Med. Oral Pathol..

[6] Walton R.E., Vertucci F.J., Walton R.E., Torabinejad M. (1996). Internal anatomy. Principles and Practice of Endodontics.

[7] Valencia de Pablo Ó., Estevez R., Peix M., Heilborn C. (2010). Root Anatomy and Canal Configuration of the Permanent Mandibular First Molar: A Systematic Review. J. Endod..

[8] Clark C.A. (1910). A method of ascertaining the relative position of unerupted teeth by means of film radiographs. Proc. R. Soc. Med..

[9] Slowey R.R. (1979). Root canal anatomy road map to successful endodontics. Dent. Clin. N. Am..

[10] Cleghorn B.M., Christie W.H., Dong C.C.S. (2007). The Root and Root Canal Morphology of the Human Mandibular First Premolar: A Literature Review. J. Endod..

[11] Scarfe W.C., Farman A.G., Sukovic P. (2006). Clinical Applications of Cone-Beam Computed Tomography in Dental Practice. J.-Can. Dent. Assoc..

[12] Miles D.A. (2005). Clinical Experience with Cone-beam Volumetric Imaging—Report of findings in 381 Cases. Comput. Tomogr..

[13] Patel S. (2009). New dimensions in endodontic imaging: Part 2. Cone beam computed tomography. Int. Endod. J..

[14] Patel S., Dawood A., Pitt T., Whaites E. (2007). The potential applications of cone beam computed tomography in the management of endodontic problems. Int. Endod. J..

[15] Sarsam W., Davies J., Al-Salehi S.K. (2025). The role of imaging in endodontics. Br. Dent. J..

[16] Shetty A., Rai S. (2026). Cone-beam computed tomography in endodontics: Historical development, technical principles, clinical applications and operative outcomes (Review). World Acad. Sci. J..

[17] Sonick M., Abrahams J., Faiella R.A. (1994). A comparison of the accuracy of periapical, panoramic, and computerized tomographic radiographs in locating the mandibular canal. Int. J. Oral Maxillofac. Implants.

[18] Shemesh A., Lalum E., Ben Itzhak J., Levy D.H., Lvovsky A., Levinson O., Solomonov M. (2020). Radicular Grooves and Complex Root Morphologies of Mandibular Premolars among Israeli Population. J. Endod..

[19] Lu T.Y., Yang S.F., Pai S.F. (2006). Complicated root canal morphology of mandibular first premolar in a Chinese population using the cross section method. J. Endod..

[20] Yu X., Guo B., Li K.Z., Zhang R., Tian Y.Y., Wang H., Hu T. (2012). Cone-beam computed tomography study of root and canal morphology of mandibular premolars in a western Chinese population. BMC Med. Imaging.

[21] Kazemipoor M., Poorkheradmand M., Rezaeian M., Safi Y. (2015). Evaluation by CBCT of root and canal morphology in mandibular premolars in an Iranian population. Chin. J. Dent. Res..

[22] Sajed M., Alvandifar S., Mallahi M. (2024). Evaluation of Root Canal Morphology of Mandibular Premolars Using Cone-beam Computed Tomography in Golestan Province, North of Iran. Iran. Endod. J..

[23] Park J.B., Kim N.R., Park S., Kim Y., Ko Y. (2013). Evaluation of permanent mandibular premolars and molars in a Korean population with cone-beam computed tomography. Eur. J. Dent..

[24] Choi Y.J., Lee C., Jeon K.J., Jang J.T., Han S.S. (2022). Canal configuration and root morphology of mandibular premolars using cone-beam computed tomography in a Korean population. Clin. Oral Investig..

[25] Bulut D.G., Kose E., Ozcan G., Sekerci A.E., Canger E.M., Sisman Y. (2015). Evaluation of root morphology and root canal configuration of premolars in the Turkish individuals using cone beam computed tomography. Eur. J. Dent..

[26] Celikten B., Orhan K., Aksoy U., Tufenkci P., Kalender A., Basmaci F., Dabaj P. (2016). Cone beam evaluation of root canal morphology of maxillary and mandibular premolars in a Turkish Cypriot population. BDJ Open.

[27] Alghamdi F.T., Khalil W.A. (2022). Root canal morphology and symmetry of mandibular second premolars using cone-beam computed tomography. Oral Radiol..

[28] Al-Zubaidi S.M., Almansour M.I., Alshammari A.S., Al Mansour N.N., Alshammari A.F., Altamimi Y.S., Madfa A.A. (2022). Root and Canal Morphology of Mandibular Premolars in a Saudi Subpopulation: A Cone-Beam Computed Tomography Study. Int. J. Dent..

[29] Mashyakhy M., Jabali A., AbuMelha A., Almasrahi M.Y., Alshahrani M.A., Alamir A., Alkahtany M., Bhandi S. (2022). Anatomical Evaluation of Mandibular Premolars in Saudi Population: An In Vivo Cone-beam Computed Tomography Study. Open Dent. J..

[30] Alsofi L., Al-Habib M., Zahran S., Alsulaiman M., Barayan M., Khawaji S., Sanari M., Altorkestani M., Alshehri L., Zarei L. (2025). Three-dimensional evaluation of root canal morphology in mandibular premolars of Saudi individuals: A CBCT study. Libyan J. Med..

[31] Almehrzi H., Khawajah S., Alharbi N., Abed R.E., Jamal M. (2025). Evaluation of the Root and Canal Morphology of Maxillary and Mandibular Premolars in an Emirati Sub-Population. Int. Dent. J..

[32] Elsayed M.A., Elmesellawy M.Y., Schäfer E. (2024). Prevalence of multiple roots and complex canal morphology in mandibular premolars among a selected Southern Egyptian sub-population: A CBCT-analysis. Odontology.

[33] Thanaruengrong P., Kulvitit S., Navachinda M., Charoenlarp P. (2021). Prevalence of complex root canal morphology in the mandibular first and second premolars in Thai population: CBCT analysis. BMC Oral Health.

[34] Datta S., Mukherjee S., Mazumdar P., Mukherjee S. (2024). An In-Vitro Study of the Root Canal Configuration of Mandibular First Premolars in a Bengali Subpopulation Using Cone-Beam Computed Tomography. Cureus.

[35] Reda R., Zanza A., Bhandi S., Biase A., Testarelli L., Miccoli G. (2022). Surgical-anatomical evaluation of mandibular premolars by CBCT among the Italian population. Dent. Med. Probl..

[36] Mrozek M., Marek E., Blum H., Pauly D., Dammaschke T., Szyszka-Sommerfeld L., Droździk A., Łagocka R., Safranow K., Lipski M. (2026). Root and canal morphology of mandibular first premolars in a Polish population: A cone-beam computed tomography study. Folia Morphol..

[37] Cleghorn B.M., Christie W.H., Dong C.C.S. (2007). The root and root canal morphology of the human mandibular second premolar: A literature review. J. Endod..

[38] Gakis P., Farmakis E.R. (2013). Endodontic considerations on the variations of the anatomy of the mandibular premolars. Saudi Endod. J..

[39] Vaghela D.J., Sinha A.A. (2013). Endodontic management of four rooted mandibular first premolar. J. Conserv. Dent..

[40] Hu P., Feng S., Li X., Li G., Li S. (2024). Endodontic treatment of a two-rooted mandibular first premolar with four root canals: A case report. Front. Dent. Med..

[41] Holtzman L. (1998). Root canal treatment of mandibular second premolar with four root canals: A case report. Int. Endod. J..

[42] Farmakis E.T. (2008). Four-rooted mandibular second premolar. Aust. Endod. J..

[43] Al-Fouzan K.S. (2001). The microscopic diagnosis and treatment of a mandibular second premolar with four canals. Int. Endod. J..

[44] Rhodes J.S. (2001). A case report of unusual anatomy: A mandibular second premolar with four canals. Int. Endod. J..

[45] Ghiasi J., Javidi M., Farhangnia A., Shayan E. (2015). Mandibular Second Premolar with Four Canals. J. Dent. Mater. Tech..

[46] Tzanetakis G.N., Lagoudakos T.A., Kontakiotis E.G. (2007). Endodontic treatment of a mandibular second premolar with four canals using operating microscope. J. Endod..

[47] Niavarzi S., Ghabraei S., Malekpour F. (2022). Mandibular Second Premolar with Four Canals: A Case Report. Iran. Endod. J..

